# Analysis of knowledge, attitudes, and practices related to antibiotics and antimicrobial resistance awareness among community members in Ghana and Burkina Faso

**DOI:** 10.1186/s13756-025-01594-7

**Published:** 2025-06-25

**Authors:** Elisenda Cama i Gibernau, Leslie Mawuli Aglanu, Alphonse Zakane, Denise Dekker, Albrecht Jahn, Ali Sié, John Humphrey Amuasi, Aurélia Souares

**Affiliations:** 1 Heidelberg Institute of Global Health, Heidelberg University Hospital, Im Neuenheimer Feld 130.3, Heidelberg, Germany; 2 German Center for Infection Research, Heidelberg Site, Germany; 3 Kumasi Centre for Collaborative Research in Tropical Medicine, Kumasi, Ghana; 4 University Medical Centre Groningen (UMCG), University of Groningen, Groningen, The Netherlands; 5 Bernhard Nocht Institute for Tropical Medicine, Hamburg, Germany; 6 Kwame Nkrumah University of Science and Technology, Kumasi, Ghana; 7 University Medical Centre Hamburg-Eppendorf, Hamburg, Germany; 8 Centre de Recherche en Santé de Nouna, Nouna, Burkina Faso

**Keywords:** Antibiotics, Antimicrobial resistance, KAP, Survey, Community members, West Africa

## Abstract

**Background:**

Antimicrobial resistance (AMR) is a global health concern, particularly in low- and middle-income countries. As human behaviour plays a crucial role in the emergence and spread of resistance, data on the understanding of AMR awareness are very important for assessing the situation and developing effective interventions. The aim of this study was to analyse the knowledge, attitudes and practices (KAP) related to antibiotics and awareness towards antibiotic resistance among community members in two districts in Ghana, and Burkina Faso.

**Methods:**

A cross-sectional survey was used to collect data on socio-demographic, economic factors, and KAP. In Burkina Faso a simple randomization was carried out, whereas in Ghana we performed a double-stage randomization. The data was collected using an electronic data capture between February and March 2023 in Ghana, and from July to November 2023 in Burkina Faso. Data analysis employed descriptive statistics, and logistic regressions.

**Results:**

A total of 1,114 participants in Ghana and 1,011 in Burkina Faso were included. The majority knew the term “Antibiotic” (Ghana: *n* = 687, 61.67%; Burkina Faso: *n* = 767, 75.87%), but only a minority were aware of AMR (Ghana: *n* = 381, 34.2%; Burkina Faso: *n* = 270, 26.71%). In both countries, participants had a middle level of knowledge about antibiotics (Ghana: *n* = 597; 53.59%, Burkina Faso: *n* = 502, 49.65%), positive attitudes towards antibiotic utilization (Ghana: *n* = 702, 63.02%; Burkina Faso: *n* = 510, 50.45%), and most of them reported a responsible use of antibiotics (Ghana: *n* = 875, 78.55%; Burkina Faso: *n* = 713, 70.52%).

**Conclusions:**

Despite familiarity with antibiotics, self-reported responsible use did not align with actual observed behaviours in both countries. Additionally, a significant lack of awareness about AMR highlights the need for a targeted educational intervention to enhance understanding of its risks and increase appropriate practices.

**Supplementary Information:**

The online version contains supplementary material available at 10.1186/s13756-025-01594-7.

## Introduction

Antimicrobial resistance (AMR) is considered a global health threat, the World Health Organization (WHO) has placed AMR among the top 10 priorities [1] as the emergence and spread of AMR pathogens threaten the ability to treat common infections and to perform life-saving procedures, such as cancer chemotherapy, caesarean section, organ transplantation and other surgeries [2]. A study in 2019 estimated approximately 5 million deaths associated with AMR, and sub-Saharan Africa has been estimated to carry the highest burden with 27.3 deaths per 100,000 habitants. In addition, the impact of AMR on health systems, the global economy and development are severe in LMICs, where limited access to healthcare, weak regulatory frameworks, and a high burden of infectious diseases exacerbate the problem [2, 3].

As AMR is a worldwide concern, different approaches have been taken to tackle this issue. In May 2015, during the 68th World Health Assembly, the “Global Action Plan on Antimicrobial Resistance” (GAP) was adopted [4] defining five main objectives: (1) strengthen knowledge, and evidence base; (2) reduce the incidence of infection; (3) optimize the use of antimicrobial medicines; (4) improve awareness and understanding of AMR and (5) develop the economic case for sustainable investment [5]. Thus, 178 countries developed their National Action Plans aligned with the objectives of the GAP [4]. Moreover, the WHO and other international organisations, launched several frameworks to support the countries in the development of their National Action Plan and to develop or strengthen their AMR surveillance systems [6].

As highlighted among the objectives of the GAP, providing evidence on knowledge and improving awareness and understanding of the looming threat of AMR is crucial to its control and prevention. Therefore, generating sufficient data on the underlying knowledge, attitudes, and practices fuelling the misuse and overuse of antibiotics is critical to promote behavioural change towards a rational and appropriate use of antibiotics [7]. Very few studies have been published on the topic in West Africa. A study conducted in Cape Coast, Ghana, found no significant differences in AMR knowledge across educational levels, although individuals working in the healthcare sector were found to have significantly higher knowledge [8]. Research in the Greater Accra and Upper West regions found that 63% of respondents were unaware of antibiotic resistance; while, 70% had taken antibiotics in the previous year, commonly using amoxicillin, amoxicillin-clavulanic acid, ampicillin, ciprofloxacin, and metronidazole [9]. In the Northern Region, in the Tamale metropolis, a study found that factors such as being female, belonging to larger households, and having higher income were associated with better antibiotic knowledge [10]. Whereas in Burkina Faso, one study was conducted among poultry farmers in Ouagadougou and revealed that 85.7% lacked knowledge about the rational use of antibiotics, only 17.1% had attended a training on poultry production, and many used antibiotics without prescriptions [11]. Another study in Burkina Faso among human health workers and veterinarians revealed that 60% had knowledge about AMR, yet 96.4% of the sample used self-medication [12].

Despite the described studies, data is largely missing for West Africa, particularly concerning the KAP of community members on a large scale, as previous research has mainly focused on specific subgroups such as healthcare workers or farmers [13]. Therefore, to contribute to the GAP and address the lack of data on knowledge and awareness of AMR, particularly in LMICs, this study aimed to develop a deeper understanding of the level of knowledge, attitudes, and practices related to antibiotics and AMR awareness among community members in Nouna District, Burkina Faso, and Asante Akim North District, Ghana.

## Methods

### Study design, setting and sample

A collaboration between research institutions from Germany, France, Burkina Faso and Ghana developed a project called AMR-B-Change (ANR/BMBF funded) with the aim to develop an intervention to promote a rational use of antibiotics and awareness about AMR. The project combined anthropological, microbiological and socio-epidemiological studies. This paper presents the data of the epidemiological component, which consisted of a cross-sectional survey conducted in two health districts: the Kossi Province in Burkina Faso and the Asante Akim North District in Ghana. The envisaged sample size was 1,000 households in each country, based on the need to achieve sufficient statistical power for subgroup analysis and to ensure demographic and geographic representativeness. In order to select the households, a double-stage random selection was performed in Ghana, it began with a proportional to size calculation to allocate number of households to be surveyed across the four sub-districts that are part of the district. In the first stage, clusters were identified within each sub-district based on community population sizes. In the second stage, households were systematically selected within each cluster by field interviewers, who followed a predefined route in their assigned area and selected every second or third household, depending on the required sample size. The starting point was randomly chosen within the area to ensure impartiality, and the selection interval (e.g., every 2nd or 3rd household) was determined in advance to maintain consistency across clusters. Whereas in Burkina Faso simple random selection was applied, as a complete list of households was available due to the presence of a Health and Demographic Surveillance System (HDSS) in the study area. The difference in selection of the households was due to the difference in data availability for household selection, and since the study did not aim to compare the two countries, analyses were performed separately for each context.

The Asante Akim North District is in the eastern part of the Ashanti region of Ghana. According to the population and housing census of 2021, this district covers an area of 1,095 km^2^ and has a total of 85,788 inhabitants, of which 56,792 live in urban area and 28,998 in rural area [14]. In Burkina Faso, the catchment area of the HDSS, is approximately 1,775 km², with a total population of 115,000 inhabitants living in 14,000 households [15]. Both study sites are predominantly rural with some semi-urban areas and were selected based on existing infrastructure and partnerships supporting AMR research. The Asante Akim North District and the Nouna District were chosen due to ongoing collaboration between KCCR and the Presbyterian Hospital in Agogo and between Nouna and the Heidelberg Institute of Global Health. In both sites an AMR surveillance system has been established since 2018 and both are part of the WHO’s Global Antimicrobial Resistance and Use Surveillance System (GLASS) network [16].

### Data collection

Data collection was performed in Ghana from mid-February 2023 until end-March 2023. Whereas in Burkina Faso from July 2023 until November 2023, due to the political instability during that period that limited access to some rural areas. Data collection was conducted by a team of 11 interviewers in Ghana, and seven in Burkina Faso. Both teams were previously trained for a total period of five days. In both settings, data was collected using a computer-assisted personal interviewing (CAPI) system with a tablet.

The survey consisted of two sections: one targeting the household head, who answered questions on socio-economic status, demographics, and antibiotic use among household livestock; the other focused, on knowledge, attitudes, and practices (KAP) related to antibiotics and awareness towards antimicrobial resistance. For the KAP section, one adult household member (> 18 years) was randomly selected from those available at the time of the interview, using a random draw and also ensuring age and gender diversity in the sample. To develop the questionnaire, we used the Multi-Country Public Awareness Survey from the World Health Organization (WHO) [17] and previous literature [8, 18, 19]. Different types of questions were used: multiple-choices, dichotomous (yes/no) and Likert scales. The last one ranged from “strongly disagree” to “strongly agree”, with five levels of response. Higher scores indicated favourable responses, with negative statements reverse-coded to maintain consistency [20]. Also, there were statements regarding antibiotics and its use to be marked as true or false. These statements covered common misconceptions, such as for example whether antibiotics were effective for treating conditions like cold/flu, sore throat, or fever.

Additionally, in order to check the validity of the questionnaire, this was pre-tested on a small sample of respondents in both countries [20]. In Ghana, 15 households and in Burkina 18 households were selected from an area outside the chosen villages but with similar characteristics. In both countries, the pre-test day lasted for one day.

### Data quality

As data collection was performed using a CAPI approach, data were directly available. Also, in order to ensure the reliability of the survey responses, a daily monitoring was performed during the data collection period, together with a weekly reporting of the data collected. This also allowed to control the inclusion of interviewees age and gender representative of the population. After checking and preliminary cleaning, the data was extracted and transferred into STATA 17 for final cleaning, codification, and analysis.

The rate of missing data was low, i.e. less than 5%, thus, deductive imputation or mean and mode imputation were performed, depending on the nature of the variable. Then, consistency checks were conducted to detect and clean errors [21]. Potential bias could be selection bias, or information bias, as these were self-reported practices [22]. Confounding was controlled by including variables, such as age, gender, comorbidities, SES, healthcare access, medicine use, ethnicity, residence, and health insurance.

Finally, to ensure high-quality presentation of the data the guideline called Strengthening the Reporting of Observational studies in Epidemiology (STROBE) was followed, because it provides a checklist to ensure a clear presentation of the observational study (Supplementary Material 7) [23].

### Statistical analysis

First, descriptive analyses were used to summarize sample characteristics using frequencies and percentages for categorical data, and means with standard deviations for linear data. Following this, some of the variables were grouped, such as socio-demographic and economic variables into different ranges or groups depending on the nature of the item. Socio-economic status (SES) was assessed through principal component analysis (PCA), using a range of variables related to household living conditions and assets. These included factors such as water source, housing type, flooring and roofing materials, toilet facilities, sources of cooking and lighting energy, housing tenure (ownership or rental), and employment status, which reflects the household’s economic engagement. All variables were recoded into binary indicators prior to inclusion in the analysis [24]. Households were subsequently ranked and grouped into quartiles, representing four levels from the lowest (Q1) to the highest SES (Q4) [24].

The level of knowledge, attitudes, and practices towards antibiotics was measured by giving a point to the correct answers, summing them up and then classifying the scores into groups. The measurement of knowledge was categorized into three groups: low, middle, and high. The total scores were categorized into three groups: low (0–4 points), middle (5–7 points), and high (8–10 points). For attitudes, each response was assigned a score based on the agreement with the statement, reflecting the type of attitude expressed: 1 for positive attitudes, 0 for negative ones, and 0.5 for neutral responses. To calculate a total attitude score, the results were summed and classified: negative (1–2.5), neutral (3–4), and positive (4.5–6). Finally, for the practices, 1 point was given for each answer that indicated a responsible use, 0 points for non-responsible use. Finally, these were sum up and categorized into two groups (0–4 points non-responsible users; 5–8 points responsible users).

Regression analyses were conducted for each country’s data. For Ghana, multinomial logistic regression was used because the outcome variables (e.g., knowledge and attitudes) had three response categories with adequate distribution across all levels. This model produces Relative Risk Ratios (RRRs), which reflect the relative probability of being in one response category versus the reference category. Although termed “relative risk” in the model output, these estimates do not imply causality or temporal relationships. In contrast, the data from Burkina Faso showed a strong imbalance across outcome categories, for example, in the knowledge variable, there were many respondents in the low and middle knowledge categories but very few in the high category. This sparsity led to convergence issues in multinomial regression. To address this, we collapsed the outcomes into binary variables and used binary logistic regression, reporting Odds Ratios (ORs). As we are not comparing the results between countries, each model was chosen based on the distribution of the data in that context, with the aim of identifying key variables associated with knowledge, attitudes, and practices.

Independent variables were residence (urban or rural), health insurance, gender, age, religion, ethnicity, literacy, employment, and socio-economic status. For Burkina Faso, religion, and ethnicity were not considered, as it was a requirement from the ethics committee. Also, health insurance was not included as frequencies were extremely low (< 5%).

Collinearity was assessed separately for each country using methods appropriate to the regression model applied. As “years in school” and “literacy” were logically correlated, their relationship was specifically examined to understand their behaviour within the models. In Burkina Faso, a high correlation was observed between the two variables. Given their overlapping contribution to the model, “years in school” was removed and “literacy” retained due to its stronger association with the outcomes. In Ghana, no significant collinearity was detected; correlation coefficients remained within acceptable thresholds, so all variables were retained in the regression analyses.

## Results

### Socio-demographic/economic factors

Participation rate in both countries was high. In Ghana 1,114 households were included (98.6%), whereas in Burkina Faso 1,011 households were included (99.7%). Socio-demographic and economic characteristics are presented in Table 1. In Ghana, the main religion was Christian (78.8%) and the main ethnicity was Akan (67.4%).

**Table 1:** Socio-demographic and economic factors in Ghana and Burkina Faso

	Ghana (*N* = 1,114)	Burkina Faso (*N* = 1,011)
Variable	**Frequency (%)**	**Frequency (%)**
**Residence**		
Semi-urban	797 (71.54)	694 (68.64)
Rural	317 (28.46)	317 (31.36)
**Gender**		
Male	474 (42.55)	606 (59.94)
Female	640 (57.45)	405 (40.06)
**Age (years)**		
18–29	432 (38.78)	304 (30.07)
30–39	303 (27.20)	203 (20.08)
40–49	167 (14.99)	208 (20.57)
> 50	212 (19.03)	296 (29.29)
**Literacy (read and write)**		
No	320 (28.73)	416 (41.15)
Yes	794 (71.27)	595 (58.85)
**Employment**		
Working (not as a farmer)	425 (38.15)	330 (32.64)
Farmer	319 (28.64)	548 (54.20)
Student	209 (18.76)	77 (7.62)
Not employed	161 (14.45)	56 (5.54)
**SES**		
Q1	279 (25.04)	253 (25.32)
Q2	278 (24.96)	254 (25.42)
Q3	279 (25.04)	252 (24.63)
Q4	278 (24.96)	252 (24.63)

### Knowledge, attitudes, and practices towards antibiotics

Participants in both countries reported to identify medicines mainly by name (Ghana: 73.3%, Burkina Faso: 66.2%), by packaging (Ghana: 45.4%, Burkina Faso: 17.7%), and for a smaller proportion by colour (Ghana: 19.9%, Burkina Faso: 15.8%). The vast majority of respondents correctly disagreed with the misconception that tablets of the same colour treat the same illness (Ghana: 83.2%, Burkina Faso: 86.6%). More than half of participants in both settings knew the word “Antibiotic” (Ghana: 61.7%, Burkina Faso: 75.9%). Also, the medicines most known were paracetamol (Ghana: 89%, Burkina Faso: 93.9%), amoxicillin (Ghana: 59.5%, Burkina Faso: 62%), and ibuprofen (Ghana: 42.7%, Burkina Faso: 60.8%).

A series of statements on antibiotic use were presented to participants. Over half of the participants in Burkina Faso (62.9%) believed antibiotics were useful for treating cold/flu, compared to only a quarter in Ghana (28.8%). Despite this difference, around half of the participants in both countries (Ghana: 54.8%, Burkina Faso: 52.5%) indicated that antibiotics should be taken for symptoms like colds, sore throats, or fevers. Regarding their attitudes, among all participants the majority agreed that parents should make sure that their children vaccination are up-to-date (Ghana: 97.8%, Burkina Faso: 97.5%), and that all individuals should wash their hands regularly (Ghana: 98.5%, Burkina Faso: 99.6%). Participants reported following doctors’ recommendations. Individuals interviewed said they would take antibiotics only when the doctor prescribed them (Ghana: 70.5%, Burkina Faso: 52%), and if the physician would not prescribe them, then they would trust the decision (Ghana: 75%, Burkina Faso: 76.5%). A lower proportion in Ghana than Burkina Faso would go to another doctor or health facility to get the antibiotic (7.1% vs. 21.9%), and more in Ghana than in Burkina Faso would try to get antibiotics without prescription (17.1% vs. 2.2%).

Financial constraints were observed in Ghana, as one third of the participants reported to take antibiotic without consulting a doctor due to lack of money (31.7%), or to the price (54.4%). Whereas in Burkina Faso, this was less reported by participants (13.3% and 24%).

Regarding the ease of obtaining antibiotics without a prescription, a higher proportion of the participants in Ghana reported that it was easy or very easy compared to the participants in Burkina Faso (88% vs. 39.7%). Nonetheless, in both countries people claimed that the sale of antibiotics should be more regulated (Ghana: 68.3%, Burkina Faso: 80.5%).

Table 2 presents the results of Knowledge, Attitudes, and Practices (KAP). In Ghana, 50.4% of participants reported knowing people who overuse antibiotics, compared to 25% in Burkina Faso Despite the reported good practices related to antibiotics. Also, around one quarter reported that they manipulate the antibiotics before taking them, i.e., adding them into food, or mixing them with milk, or alcohol (Ghana: 30.7%, Burkina Faso: 37.5%).

**Table 2:** Level of knowledge, attitudes, and practices in Ghana and Burkina Faso

	Ghana (*N* = 1,114)	Burkina Faso (*N* = 1,011)
Variable	**Frequency (%)**	**Frequency (%)**
**Knowledge**		
Low	210 (18.85)	463 (45.80)
Middle	597 (53.59)	502 (49.65)
High	307 (27.56)	46 (4.55)
**Attitudes**		
Negative	43 (3.86)	13 (1.29)
Neutral	369 (33.12)	488 (48.27)
Positive	702 (63.02)	510 (50.44)
**Practices**		
Non – responsible users	239 (21.45)	298 (29.48)
Responsible users	875 (78.55)	713 (70.52)

Several factors were significantly associated with the level of knowledge about antibiotics. In Ghana, individuals living in semi-urban areas were less likely to have a low level of knowledge (RRR = 0.44 [95% CI: 0.30–0.65]), indicating that semi-urban residency is associated with higher knowledge levels. Similarly, having more years of schooling was associated with a lower likelihood of low knowledge (RRR = 0.38 [95% CI: 0.17–0.83]), highlighting the positive impact of education. Positive factors influencing higher knowledge included being literate (RRR = 0.32 [95% CI: 0.15–0.68]), being married (RRR = 0.49 [95% CI: 0.32–0.75]), belonging to the Ewe ethnic group (RRR = 0.21 [95% CI: 0.10–0.41]), and belonging to a higher socioeconomic status (Q3: RRR = 0.46 [0.26–0.79]; Q4: RRR = 0.42 [95% CI: 0.24–0.74]). These groups were less likely to have low knowledge levels, suggesting that literacy, marital status, ethnicity, and socioeconomic status positively influence antibiotic knowledge. Conversely, participants over 50 years old were more likely to have a low level of knowledge (RRR = 1.95 [95% CI: 1.04–3.66]). Those practicing traditional religion (RRR = 10.36 [95% CI: 2.31–46.35]) or no religion (RRR = 3.59 [95% CI: 1.47–8.75]) also had a higher risk of low knowledge levels. These findings identify older age and certain religious affiliations as risk factors for lower knowledge about antibiotics (Supplementary Material 1). In Burkina Faso, participants were more likely to have a higher level of knowledge when being literates, i.e., reading and speaking French (aOR = 1.45 [95% CI: 1.08–1.94]), semi-urban residency (aOR = 2.79 [95% CI: 1.84–4.24]), and higher SES (Q2 aOR = 2.44 [95% CI: 1.59–3.74]; Q3 aOR = 1.86 [95% CI: 1.13–3.06; Q4 aOR = 2.55 [95% CI: 1.51–4.33]) (Supplementary Material 4).

In Ghana, participants residing in semi-urban areas were less likely to report negative attitudes compared to those in rural areas (RRR = 0.46 [95% CI: 0.23–0.93]), indicating that living in semi-urban areas is associated with more positive attitudes. Conversely, participants practicing traditional religions were significantly more likely to hold negative attitudes toward antibiotics (RRR = 12.57 [95% CI: 2.58–61.28]) (Supplementary Material 2). In the context of Burkina Faso, literacy and being a student were factors influencing significantly positive attitudes toward antibiotics. Literate individuals were almost twice as likely to report positive attitudes compared to neutral or negative ones (aOR = 1.68 [95% CI: 1.21–2.18]). Similarly, students were nearly two times more likely to show positive attitudes (aOR = 1.93 [95% CI: 1.08–3.46]). These findings suggest that education in general is associated with more favourable attitudes toward antibiotic use in Burkina Faso (Supplementary Material 5).

Factors influencing the non-responsible use of antibiotics in Ghana were: living in semi-urban areas (RRR = 2.38 [95% CI: 1.63–3.45]), and no religious affiliation (RRR = 3.23 [95% CI: 1.54–6.75]). On the other hand, having health insurance was a factor associated with a responsible use of antibiotics (RRR = 0.65 [95% CI: 0.48–0.89]) (Supplementary Material 3). Whereas in Burkina Faso, factors influencing responsible antibiotic use in Burkina Faso included living in semi-urban areas (aOR = 2.60 [95% CI: 1.66–4.08]) and higher socio-economic status (Q2: aOR = 2.80 [95% CI: 1.81–4.32]; Q3: aOR = 2.48 [95% CI: 1.45–4.23]; Q4: aOR = 1.86 [95% CI: 1.06–3.26]). On the other hand, being unemployed was associated with a lower likelihood of responsible antibiotic use (aOR = 0.51 [95% CI: 0.26–0.97]). Literacy, which was significant in the unadjusted model (OR = 1.51 [95% CI: 1.15–1.99]), lost significance after adjusting for confounders (aOR = 1.31 [95% CI: 0.95–1.79]). Similarly, being a farmer was initially linked to non-responsible antibiotic use (OR = 0.58 [95% CI: 0.42–0.79]) but was no longer significant in the adjusted model (aOR = 1.04 [95% CI: 0.71–1.51]). These results highlight the role of socio-economic factors and residency in shaping antibiotic use practices in Burkina Faso, however it is important to note that residency and SES were confounders. This confounding effect was accounted for in the analysis, and both variables were retained in the model due to their relevance in assessing antibiotic use practices (Supplementary Material 6).

### Awareness towards AMR

Among all participants a low proportion were aware of AMR (Ghana: 34.2%, Burkina Faso: 26.7%). Among those who answered knowing about resistance (Ghana: *N* = 381; Burkina Faso: *N* = 270) a sub-set of questions was asked. First, they reported where they heard of AMR; it was mainly from doctors or nurses (Ghana: 61.9%, Burkina Faso: 57.8%) and family members or friends (Ghana: 40.7%, Burkina Faso: 30.7%). In Ghana pharmacists also played a role informing about AMR (33.0%), whereas in Burkina Faso it was more media, such as TV, and newspapers (30%). In both settings specific campaigns were rarely mentioned (Ghana: 1.3%, Burkina Faso: 2.2%).

Questions regarding the transmission of antimicrobial resistance were also asked. While in both countries it seemed clear that AMR could be transmitted from animals to humans (Ghana: 64.3%, Burkina Faso: 66.7%), transmission from the environment to people was slightly more recognized in Burkina Faso (50.6%) than in Ghana (44.2%). On the contrary, participants from Ghana were more aware of the transmission of resistant bacteria from person to person (78.7%) than in Burkina Faso (39.3%).

Additionally, in both settings participants believed that they were not at risk of getting antibiotic resistant infections, as long as they would take the antibiotics correctly (Ghana: 73%, Burkina Faso: 90.4%). But still, they were worried of the impact AMR could have on their health (Ghana: 86.6%, Burkina Faso: 96.7%).

**Fig. 1:**
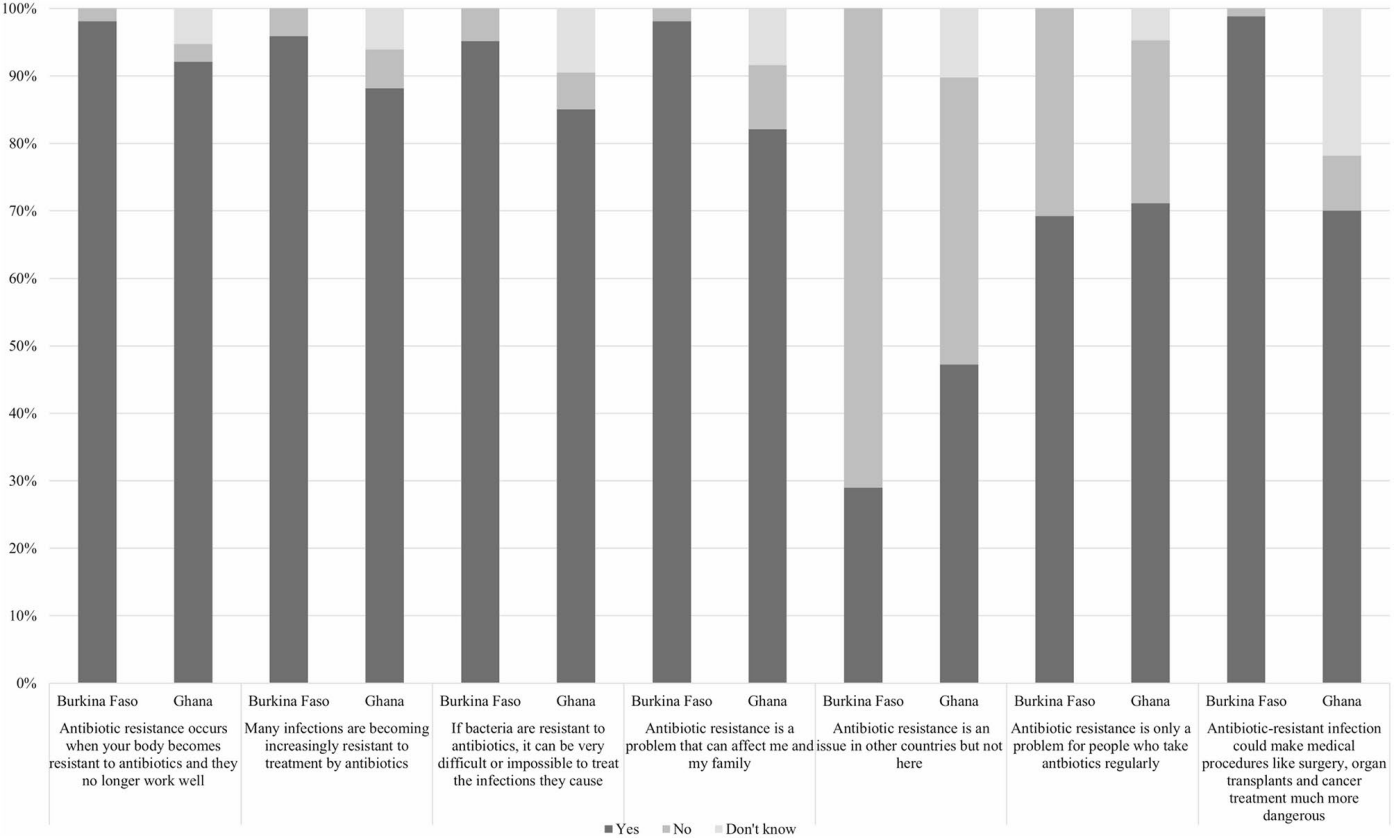
Statements regarding AMR, results from Ghana and Burkina Faso. Statements are based on the WHO Antibiotic Resistance: Multi-Country Public Awareness Survey (2015). The figure presents the proportion of participants who answered “Yes,” “No,” or “Don’t know” to each statement regarding AMR

A series of statements regarding knowledge of AMR were asked, and the results are presented in Fig. 1 to descriptively illustrate responses within each country.

## Discussion

Providing data on antibiotic knowledge, attitudes, practices, and AMR awareness is crucial to identifying gaps in public understanding and informing the development of targeted interventions to address irrational antibiotic use and combat the growing threat of AMR [5]. The results presented here have been collected through a cross-sectional survey in two semi-rural districts of Ghana and Burkina Faso.

In both countries, higher levels of antibiotic knowledge were associated with semi-urban residency, literacy, and higher socio-economic status (SES). These findings align with a study from Northern Ghana, which also identified SES, literacy, and semi-urban residency as key determinants of good antibiotic knowledge [10]. Factors associated with attitudes and practices were different in each country. In Ghana, semi-urban residency was associated with more positive attitudes toward antibiotic use, yet, it was also linked to non-responsible practices. This may be due to greater access to medicines in the private sector in semi-urban areas, which can facilitate the self-medications and inappropriate antibiotic use. Another study in the East Region of Ghana discussed inappropriate use due to easy access to antibiotics over-the-counter with or without prescription [25]. Whereas having medical insurance in Ghana was a significant predictor of responsible antibiotic use, consistent with previous studies [25, 26]. This could be explained by insured individuals having easier access to healthcare facilities, reducing the reliance on self-medication or informal antibiotic purchases. In Burkina Faso, the factor of holding a medical insurance was not observed due to the low prevalence of medical insurance among participants, a similar limitation was highlighted in another study in the same country [27]. The lack of widespread insurance coverage may result in a greater dependence on out-of-pocket medication purchases, thus contributing to non-responsible antibiotic use. Also, in Burkina Faso, literacy was a key factor for positive attitudes toward antibiotics, while higher SES and semi-urban residency were associated with responsible antibiotic practices. This may be because access to drug sellers in semi-urban is limited, which could reduce irrational antibiotic use. Nonetheless, it is important to note that the reported responsible antibiotic use in both countries was based on self-reported practices.

In general, in both settings, medicines were identified mainly by name, packaging, and colour. This uniformity in identification practices across the settings is particularly interesting because previous studies in Ghana have also highlighted medication identification based on colour or by using alternative local terminology, such as “Topaye”, which refers to medicines in capsules perceived to treat pain associated with diseases in Twi (Akan dialect spoken in this region of Ghana) [28]. In Burkina Faso, there was no specific research or detailed information on the identification of medicines; more studies about the understanding of antimicrobial drugs and local terminology would be interesting because of the representation conditions of the use of medication.

Moreover, in both countries, many participants reported using antibiotics to treat the common cold, despite most upper respiratory tract infections being viral in origin and therefore not responsive to antibiotics, which is particularly concerning given that respiratory infections remain among the top ten causes of death in both countries [29–31]. This highlights the importance of appropriate utilisation of antibiotics. It is needed to find solutions to ensure that patients who truly need antibiotics receive them, and that unnecessary prescriptions are avoided by improving diagnostic capabilities that ensure adequate prescription, and implementing interventions that increase health care workers and public awareness about proper antibiotic utilization [29–31].

Furthermore, this study found that amoxicillin was among the most recognized medicines by participants, alongside with ibuprofen and paracetamol. This is significant because amoxicillin is one of the top five most consumed antimicrobials globally [32] and is classified by the WHO as a highly important antibiotic for human and veterinary medicine [33, 34]. Despite regulations in Ghana that restrict Licensed Chemical Sellers (LCS) from selling antibiotics other than cotrimoxazole suspension, many still sell antibiotics like amoxicillin due to public demand and perceived effectiveness [35]. Similar patterns of antibiotic sales through peddlers or at markets have been observed in rural Burkina Faso [36]. The widespread recognition and misuse of amoxicillin underscore the urgent need for public education on proper antibiotic use, stricter enforcement of regulations on antibiotic sales. Weak regulatory frameworks can also be observed in the reported easiness of purchasing antibiotics particularly in Ghana. Previous research also found that participants indicated that antibiotics in this country could be obtained without a prescription in pharmacies, LCS, and drug outlets [35, 37]. Regarding the results obtained in Burkina Faso, participants claimed that accessing to antibiotics without prescription was difficult but not impossible. This is confirmed by a study in Burkina Faso that described drug peddlers and sellers in the markets, which facilitated self-medication, as accessing antibiotics through formal healthcare channels was often difficult due to geographic and economic barriers, leading to a reliance on informal sources [36]. Also, it is important to consider the current political situation, as data was collected in 2023 under political and economic instability. Research during this period has found that there was insufficient medicine stock, and a procurement monopoly [38], which may have impacted the purchase and access to antibiotics.

Additionally, regarding practices towards antibiotics, prior studies in Ghana and Burkina Faso have shown that patients do not properly adhere to medical professionals’ advice, often resulting in irrational and inappropriate use of antibiotics [28, 39, 40]. This can be observed at the level of antibiotics procurement or adherence to treatment. According to the participants in our study, they usually follow doctor’s instructions, and very few highlighted that they would seek for a specific antibiotic if not prescribed by the doctor. Patients might miscomprehend recommendations given by doctors, or doctors could not give clear explanations, even when both parties have good intentions [39–42]. This calls for improved communication strategies that will enable patients to fully understand treatment plans.

Another reported practice important to highlight is the manipulation of antibiotics, mixing them with milk, alcohol, or food. These practices were observed in both settings, and have also been reported in a systematic review and meta-analysis in West Africa, which noted that this leads to a reduced efficacy of treatment and potential increased impact on resistance development as well as side effects for the patients. Thus, it has to be considered as well when designing interventions and information and communication with the patients [43].

After the assessment of AMR awareness, the results revealed that only about a third of the total participants surveyed were aware of AMR, from which very few reported having learnt about it through awareness campaigns. As the poor level of AMR awareness among the general population has been well documented by other studies [43–45], this highlights the need of an intervention to sensitize people about this matter [44, 45].

Finally, although participants understood that bacterial infections could be transmitted from animals to humans, many believed that resistance occurs in the body itself, and it is only a problem for those who take antibiotics regularly and it does not pose a risk to them. According to the participants, AMR is considered as a health challenge in other settings or countries but not a major health issue in Ghana or Burkina Faso. This highlights the importance of targeted interventions to increase community knowledge and awareness on the mechanisms of AMR while emphasising the risks related to it. These interventions have to consider local beliefs, concepts and behaviours [46].

### Strengths and limitations

This study is the first to conduct a large-scale cross-sectional survey examining the level of knowledge, attitudes, practices towards antibiotics, and awareness of AMR among community members. This study followed a rigorous methodology, which included a robust survey design, a representative and large sample, an experienced research teams, and an extensive use of quality control measures to ensure validity and reliability of the findings. Additionally, the study captures community-level perspectives on AMR and antibiotic practices, providing insights directly relevant for the development of interventions. Furthermore, the study contributes to inform global and regional AMR action plans, aligning with the WHO’s Global Action Plan on AMR by addressing knowledge gaps at the community level. However, limitation that this study faced was the political situation that Burkina Faso is undergoing, which delayed the data collection period and may have impacted some of the results, about access to antibiotics for example. Also, data on ethnicity and religion were excluded from the analysis by the ethics committee from Burkina Faso.

## Conclusion

This KAP analysis reveals that participants in Ghana and Burkina Faso generally scored a moderate knowledge, positive attitudes, and responsible practices regarding antibiotics. Despite these trends, awareness of AMR was low, with most participants unaware of the risks AMR poses or the role of improper antibiotic use in its development and spread. Furthermore, common misconceptions, such as using antibiotics for viral infections and the widespread availability of antibiotics without prescription, highlight the need for targeted interventions. Strengthening public education by addressing knowledge gaps, promoting awareness about AMR, and enforcing stricter regulations on antibiotic sales are essential steps to combat this growing threat in these regions.

## Electronic supplementary material

Below is the link to the electronic supplementary material.

Supplementary Material 1

Supplementary Material 2

Supplementary Material 3

Supplementary Material 4

Supplementary Material 5

Supplementary Material 6

Supplementary Material 7

## Data Availability

No datasets were generated or analysed during the current study.

## Abbreviations

AMR: Antimicrobial resistance
AWaRe: Access, watch, reserve
CAPI: Computer-assisted personal interviewing
GAP: Global action plan
KAP: Knowledge, attitudes and practices
LCS: Licensed chemical shops
LMICs: Low- and middle-income countries
PCA: Principal component analysis
SES: Socio-economic status
STROBE: Strengthening the reporting of observational studies in epidemiology
WHO: World health organization
