# Supplementary material for: Analysis of knowledge, attitudes, and practices related to antibiotics and antimicrobial resistance awareness among community members in Ghana and Burkina Faso
Source: Antimicrob Resist Infect Control. 2025 Jun 25;14:72. doi: 10.1186/s13756-025-01594-7 (PMC12199504; doi:10.1186/s13756-025-01594-7)
Supplement: Supplementary file 2 — Supplementary Material 2 [file 13756_2025_1594_MOESM2_ESM.docx]

Supplementary Material 2. Multinomial regression analysis of independent variables and attitudes in Ghana

| **Attitudes** | **RRR.** | **St. Err.** | **t-value** | **p-value** | **[95% Conf** | **Interval]** | **Significance** |
| --- | --- | --- | --- | --- | --- | --- | --- |
| **Negative** |  |  |  |  |  |  |  |
| **Residence**  Ref: Rural |  |  |  |  |  |  |  |
| Semi-urban | 0.46 | 0.16 | -2.15 | 0.03 | 0.23 | 0.93 | ** |
| **Religion**  Ref: Muslim |  |  |  |  |  |  |  |
| Christian | 0.97 | 0.41 | -0.05 | 0.95 | 0.42 | 2.25 |  |
| Traditional | 12.57 | 10.16 | 3.13 | 0.00 | 2.58 | 61.28 | *** |
| No religion | 0.80 | 0.88 | -0.20 | 0.84 | 0.09 | 6.96 |  |
| **Years in school**  Ref: Never |  |  |  |  |  |  |  |
| <12 years | 0.61 | 0.24 | -1.21 | 0.22 | 0.27 | 1.35 |  |
| >12 years | 0.25 | 0.13 | -2.56 | 0.01 | 0.08 | 0.72 | ** |
| **SES**  Ref: Q1 |  |  |  |  |  |  |  |
| Q2 | 0.69 | 0.29 | -0.85 | 0.39 | 0.30 | 1.60 |  |
| Q3 | 0.41 | 0.21 | -1.67 | 0.09 | 0.14 | 1.16 | * |
| Q4 | 1.03 | 0.56 | 0.06 | 0.95 | 0.35 | 3.03 |  |
| **Neutral** |  |  |  |  |  |  |  |
| **Residence**  Ref: Rural |  |  |  |  |  |  |  |
| Semi-urban | 0.49 | 0.07 | -4.43 | 0.00 | 0.36 | 0.67 | *** |
| **Religion**  Ref: Muslim |  |  |  |  |  |  |  |
| Christian | 1.07 | 0.20 | 0.38 | 0.70 | 0.74 | 1.56 |  |
| Traditional | 3.17 | 1.99 | 1.84 | 0.06 | 0.92 | 10.88 | * |
| No religion | 3.08 | 1.10 | 3.13 | 0.00 | 1.52 | 6.24 | *** |
| **Years in school**  Ref: Never |  |  |  |  |  |  |  |
| <12 years | 0.84 | 0.17 | -0.83 | 0.40 | 0.56 | 1.25 |  |
| >12 years | 0.78 | 0.17 | -1.06 | 0.28 | 0.51 | 1.22 |  |
| **SES**  Ref: Q1 |  |  |  |  |  |  |  |
| Q2 | 0.59 | 0.11 | -2.69 | 0.00 | 0.40 | 0.86 | *** |
| Q3 | 0.41 | 0.08 | -4.12 | 0.00 | 0.27 | 0.63 | *** |
| Q4 | 1.27 | 0.27 | 1.14 | 0.25 | 0.84 | 1.93 |  |
| **Positive** (base outcome) |  |  |  |  |  |  |  |

RRR = Relative Risk Ratio; St. Err. = Standard Error; t-value = Test Statistic; p-value = Probability Value; [95% Conf Interval] = 95% Confidence Interval. The "Positive (base outcome)" refers to the reference category used in the multinomial logistic regression analysis. The RRRs for other categories (e.g., Negative, Neutral) are interpreted relative to this base outcome. Significance levels: *p < 0.05, **p < 0.01, **p < 0.001
