# Supplementary material for: Analysis of knowledge, attitudes, and practices related to antibiotics and antimicrobial resistance awareness among community members in Ghana and Burkina Faso
Source: Antimicrob Resist Infect Control. 2025 Jun 25;14:72. doi: 10.1186/s13756-025-01594-7 (PMC12199504; doi:10.1186/s13756-025-01594-7)
Supplement: Supplementary file 3 — Supplementary Material 3 [file 13756_2025_1594_MOESM3_ESM.docx]

Supplementary Material 3. Multinomial regression analysis of independent variables and practices in Ghana

| **Practices** | **RRR** | **St.Err.** | **t-value** | **p-value** | **[95% Conf** | **Interval]** | **Significance** |
| --- | --- | --- | --- | --- | --- | --- | --- |
| **Non-responsible users** |  |  |  |  |  |  |  |
| **Residence**  Ref: Rural |  |  |  |  |  |  |  |
| Semi-urban | 2.37 | 0.45 | 4.54 | 0.00 | 1.63 | 3.45 | *** |
| **Medical insurance**  Ref: No |  |  |  |  |  |  |  |
| Yes | 0.65 | 0.10 | -2.65 | 0.00 | 0.48 | 0.89 | *** |
| **Religion**  Ref: Muslim |  |  |  |  |  |  |  |
| Christian | 1.47 | 0.34 | 1.69 | 0.09 | 0.94 | 2.32 | * |
| Traditional | 2.68 | 1.53 | 1.73 | 0.08 | 0.87 | 8.23 | * |
| No religion | 3.23 | 1.21 | 3.12 | 0.00 | 1.54 | 6.75 | *** |
| **Responsible users** (base outcome) |  |  |  |  |  |  |  |

RRR = Relative Risk Ratio; St. Err. = Standard Error; t-value = Test Statistic; p-value = Probability Value; [95% Conf Interval] = 95% Confidence Interval. The "Responsible users (base outcome)" refers to the reference category used in the multinomial logistic regression analysis. The RRRs for other categories (e.g., Non-responsible users) are interpreted relative to this base outcome. Significance levels: *p < 0.05, **p < 0.01, **p < 0.001
