# Supplementary material for: Analysis of knowledge, attitudes, and practices related to antibiotics and antimicrobial resistance awareness among community members in Ghana and Burkina Faso
Source: Antimicrob Resist Infect Control. 2025 Jun 25;14:72. doi: 10.1186/s13756-025-01594-7 (PMC12199504; doi:10.1186/s13756-025-01594-7)
Supplement: Supplementary file 4 — Supplementary Material 4 [file 13756_2025_1594_MOESM4_ESM.docx]

Supplementary Material 4. Binomial logistic regression on independent variables and knowledge in Burkina Faso

| **Variables** | **Unadjusted** | | **Adjusted** | |
| --- | --- | --- | --- | --- |
|  | **OR**  **(95% CI)** | **p** | **OR**  **(95% CI)** | **p** |
| **Residence**  Ref: Rural |  |  |  |  |
| Semi-urban | 4.68  (3.51 – 6.25) | 0.00*** | 2.79  (1.84 - 4.24) | 0.00*** |
| **Literacy (read and write)**  Ref: No |  |  |  |  |
| Yes | 1.70  (1.32 – 2.20) | 0.00*** | 1.45  (1.08 - 1.94) | 0.01** |
| **Employment**  Ref: Working (not as a farmer) |  |  |  |  |
| Farmer | 0.52  (0.39 - 0.69) | 0.00*** | 1.01  (0.73 - 1.41) | 0.91 |
| Student | 1.16  (0.69 - 1.96) | 0.56 | 1.45  (0.82 – 2.57) | 0.19 |
| Not working | 0.85  (0.47 - 1.51) | 0.58 | 1.41  (0.74 – 2.68) | 0.29 |
| **SES**  Ref: Q1 |  |  |  |  |
| Q2 | 3.81  (2.62 - 5.53) | 0.00*** | 2.44  (1.59 – 3.74) | 0.00*** |
| Q3 | 4.26  (2.92 - 6.21) | 0.00*** | 1.86  (1.13 – 3.06) | 0.01** |
| Q4 | 6.54  (4.43 - 9.64) | 0.00*** | 2.55  (1.51 – 4.33) | 0.00*** |

OR = Odds Ratio; 95% CI = 95% Confidence Interval. Significance levels: *p < 0.05, **p < 0.01, **p < 0.001
