# Supplementary material for: Analysis of knowledge, attitudes, and practices related to antibiotics and antimicrobial resistance awareness among community members in Ghana and Burkina Faso
Source: Antimicrob Resist Infect Control. 2025 Jun 25;14:72. doi: 10.1186/s13756-025-01594-7 (PMC12199504; doi:10.1186/s13756-025-01594-7)
Supplement: Supplementary file 5 — Supplementary Material 5 [file 13756_2025_1594_MOESM5_ESM.docx]

Supplementary Material 5. Binomial logistic regression on independent variables and attitudes in Burkina Faso

| **Variables** | **Unadjusted** | | **Adjusted** | |
| --- | --- | --- | --- | --- |
|  | **OR**  **(95% CI)** | **p** | **OR**  **(95% CI)** | **p** |
| **Age**  Ref: 18 – 29 years |  |  |  |  |
| 30-39 years | 1.03  (0.72 – 1.47) | 0.87 | 1.34  (0.90 - 1.97) | 0.14 |
| 40-49 years | 0.66  (0.46 - .94) | 0.02** | 0.94  (0.63 - 1.39) | 0.77 |
| 50-59 years | 0.76  (0.50 - 1.14) | 0.18 | 1.13  (0.72 - 1.77) | 0.57 |
| >60 years | 0.66  (0.45 - 0.97) | 0.03** | 1.04  (0.66 - 1.65) | 0.84 |
| **Literacy (read and write)**  Ref: No |  |  |  |  |
| Yes | 1.77  (1.37 - 2.28) | 0.00*** | 1.62  (1.21- 2.18) | 0.00*** |
| **Employment**  Ref: Working (not as a farmer) |  |  |  |  |
| Farmer | 0.81  (0.62 - 1.07) | 0.14 | 0.92  (0.69 - 1.22) | 0.57 |
| Student | 2.02  (1.19 - 3.44) | 0.00*** | 1.93  (1.08 - 3.46) | 0.02** |
| Not working | 0.85  (0.48 - 1.50) | 0.58 | 1.10  (0.59 - 2.03) | 0.75 |

OR = Odds Ratio; 95% CI = 95% Confidence Interval. Significance levels: *p < 0.05, **p < 0.01, **p < 0.001
