# Supplementary material for: Analysis of knowledge, attitudes, and practices related to antibiotics and antimicrobial resistance awareness among community members in Ghana and Burkina Faso
Source: Antimicrob Resist Infect Control. 2025 Jun 25;14:72. doi: 10.1186/s13756-025-01594-7 (PMC12199504; doi:10.1186/s13756-025-01594-7)
Supplement: Supplementary file 6 — Supplementary Material 6 [file 13756_2025_1594_MOESM6_ESM.docx]

Supplementary Material 6. Binomial logistic regression on independent variables and practices in Burkina Faso

| **Variables** | **Unadjusted** | | **Adjusted** | |
| --- | --- | --- | --- | --- |
|  | **OR**  **(95% CI)** | **p** | **OR**  **(95% CI)** | **p** |
| **Residence**  Ref: Rural |  |  |  |  |
| Semi-urban | 4.10  (3.07 - 5.47) | 0.00*** | 2.60  (1.66- 4.08) | 0.00*** |
| **Literacy (read and write)**  Ref: No |  |  |  |  |
| Yes | 1.51  (1.15 - 1.99) | 0.00*** | 1.31  (0.95 - 1.79) | 0.09* |
| **Employment**  Ref: Working (not as a farmer) |  |  |  |  |
| Farmer | 0.58  (0.42 - 0.79) | 0.00*** | 1.04  (0.71 - 1.51) | 0.82 |
| Student | 0.94  (0.52 - 1.70) | 0.85 | 1.14  (0.60 - 2.16) | 0.68 |
| Not working | 0.38  (0.21 - 0.69) | 0.00*** | 0.51  (0.26 - 0.97) | 0.04** |
| **SES**  Ref: Q1 |  |  |  |  |
| Q2 | 4.18  (2.85 - 6.13) | 0.00*** | 2.80  (1.81 - 4.32) | 0.00*** |
| Q3 | 5.24  (3.50 - 7.83) | 0.00*** | 2.48  (1.45 - 4.23) | 0.00*** |
| Q4 | 4.47  (3.02 - 6.60) | 0.00*** | 1.86  (1.06 - 3.26) | 0.02** |

OR = Odds Ratio; 95% CI = 95% Confidence Interval. Significance levels: *p < 0.05, **p < 0.01, **p < 0.001
